# Supplementary material for: An Implantable Device that Converses with Patients and Learns to Co-Manage Epilepsy
Source: medRxiv. 2026 Mar 9:2026.01.26.26344234. Originally published 2026 Jan 27. Preprint. [Version 2] doi: 10.64898/2026.01.26.26344234 (PMC12870640; doi:10.64898/2026.01.26.26344234)
Supplement: 1 [file NIHPP2026.01.26.26344234V2-supplement-1.pdf]

medRxiv preprint doi: https://doi.org/10.64898/2026.01.26.26344234; this version posted March 9, 2026. The copyright holder for this preprint (which was not certified by peer review) is the author/funder, who has granted medRxiv a license to display the preprint in perpetuity.
It is made available under a CC-BY 4.0 International license .

Supplemental Materials

Supplementary methods

EEG artifact rejection

For each one-second EEG segment, we detected bad channels using a custom algorithm. Channels were considered bad and excluded from further analyses if: (1) more than 50% of samples were missing or zero-valued, (2) more than 50% of samples were flat-lined, (3) absolute deviations from the channel-wise median exceeded 5 mV for more than 10 samples, (4) extreme outliers were present, defined as values falling outside the envelope defined by the median $\pm 10 \times$ (99th percentile - median), consistent with transient disconnection or motion artifacts, and (5) power in the 58–62 Hz band exceeded 10% of total spectral power.

medRxiv preprint doi: https://doi.org/10.64898/2026.01.26.26344234; this version posted March 9, 2026. The copyright holder for this preprint (which was not certified by peer review) is the author/funder, who has granted medRxiv a license to display the preprint in perpetuity.
It is made available under a CC-BY 4.0 International license .

Supplementary figures

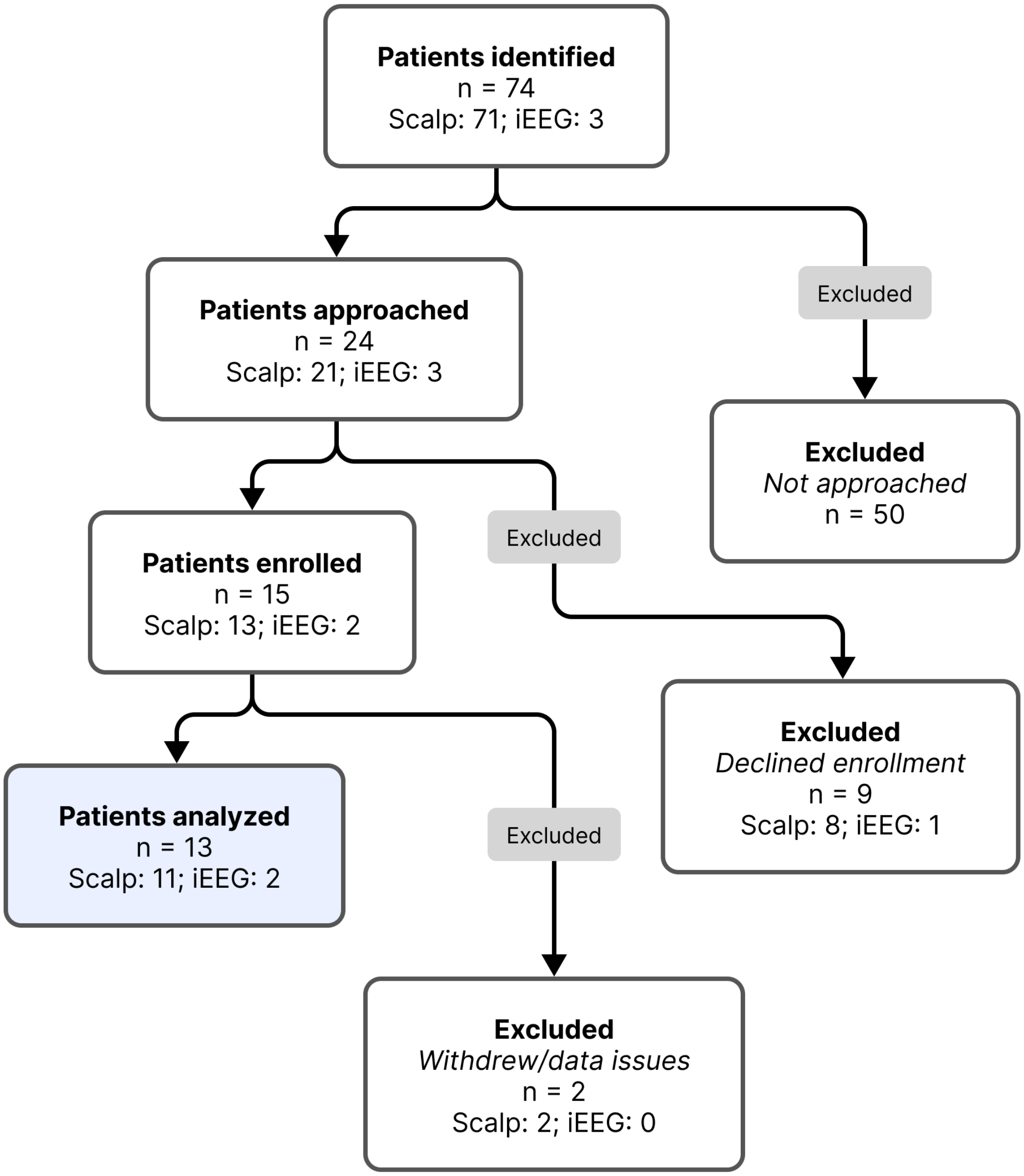

Figure S1: Patient enrollment and analysis flowchart.

medRxiv preprint doi: https://doi.org/10.64898/2026.01.26.26344234; this version posted March 9, 2026. The copyright holder for this preprint
(which was not certified by peer review) is the author/funder, who has granted medRxiv a license to display the preprint in perpetuity.
It is made available under a CC-BY 4.0 International license .

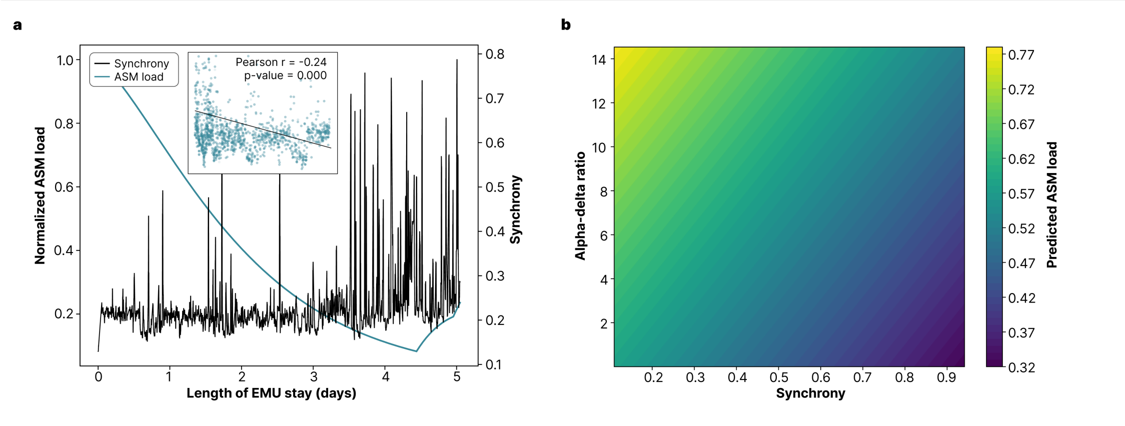

Figure S2: Synchrony as a biomarker for anti-seizure medication load. **a.** Time series of synchrony and normalized anti-seizure medication (ASM) load across the entire epilepsy monitoring unit (EMU) stay for a representative patient. **b.** Predicted response surface from a linear mixed model with normalized ASM load as the dependent variable, synchrony and alpha–delta ratio as fixed-effect predictors, and patient as a random effect.

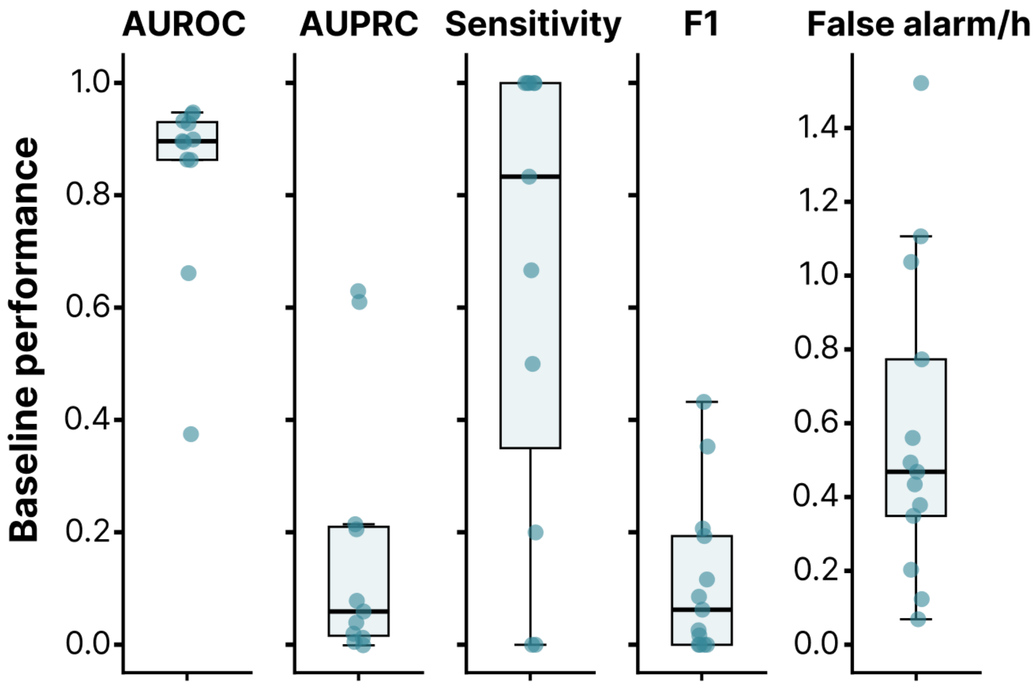

Figure S3: Seizure detection performance at baseline. Each dot represents one patient. The box represents the interquartile range (IQR) with a central line indicating the median across patients; whiskers extend to 1.5×IQR.

Abbreviations: AUROC: area under the receiver operating characteristic curve; AUPRC: area under the precision-recall curve.

medRxiv preprint doi: https://doi.org/10.64898/2026.01.26.26344234; this version posted March 9, 2026. The copyright holder for this preprint (which was not certified by peer review) is the author/funder, who has granted medRxiv a license to display the preprint in perpetuity.
It is made available under a CC-BY 4.0 International license .

Supplementary tables

Table S1: Summary of quantitative EEG features and detection algorithms.

Domain	Feature/model	Window	Modality[a]	Preprocessing requirement	Missing data handling
Sleep	Alpha-delta ratio	1 min	Both	CAR	Discard invalid
Sleep	YASA	10 min[b]	Both[c]	CAR	Discard if <5min valid
ASM estimation	Synchrony	1 min	Both	CAR	Drop window
	ASM load	1 min	Both	N/A	N/A
Spike detection	SpikeNet	2s (1s shift)	Scalp	CAR and bipolar	Drop window
	Spike detector	1 min	iEEG	CAR	Drop window
Seizure detection	SPaRCNet	10s (2s shift)	Scalp	Bipolar	Drop window
	WaveNet	1s	iEEG	Bipolar prewhitened	Drop window
	ONCET	1s	iEEG	Bipolar prewhitened	Drop window

Abbreviations: CAR: common average re-reference; Bipolar: bipolar re-reference; YASA: Yet Another Spindle Algorithm; ASM: anti-seizure medication; iEEG: intracranial EEG.
[a] Whether the feature or model is applied on scalp or intracranial EEG.
[b] Calculated at 30s intervals.
[c] Requires scalp channels C3, C4, or Cz.

Table S2: Event definitions and post-processing criteria.

Event	Source model	Criteria; Additional metrics
Seizure detection	SPaRCNet (scalp)	Duration $\geq$ 10s (gap $\leq$ 2s); Mean prob $\geq$ 0.8, max $\geq$ 0.95
	WaveNet (iEEG)	Duration $\geq$ 10s (gap $\leq$ 2s)
	ONCET (iEEG)	Duration $\geq$ 10s (gap $\leq$ 2s)
Elevated spike rate	SpikeNet (scalp)<br>Spike detector (iEEG)	Duration $\geq$ 1 min (gap $\leq$ 1 min); Mean rate $\geq$ 10 spikes/min
Sleep report	YASA	12h blocks (7:00–19:00 / 19:00–7:00); Absolute duration and proportion of each stage

Abbreviations: iEEG: intracranial EEG; YASA: Yet Another Spindle Algorithm.

medRxiv preprint doi: https://doi.org/10.64898/2026.01.26.26344234; this version posted March 9, 2026. The copyright holder for this preprint (which was not certified by peer review) is the author/funder, who has granted medRxiv a license to display the preprint in perpetuity.
It is made available under a CC-BY 4.0 International license .

Table S3: Morning survey (8:00–9:00).

No.	Question	Construct	Anchors (VAS 1–100)
1	Right now, how sad do you feel?	Depressed mood	Not at all – Extremely
2	Right now, how anxious do you feel?	Anxiety	Not at all – Extremely
3	Right now, how sharp is your thinking?	Cognition	Not at all – Extremely
4	Right now, how stressed do you feel?	Stress	Not at all – Extremely
5	Right now, how fatigued do you feel?	Fatigue	Not at all – Extremely
6	Right now, how hopeful do you feel about things?	Depressed mood	Not at all – Extremely
7	Right now, how tense do you feel?	Anxiety	Not at all – Extremely
8	Right now, how difficult is concentrating?	Cognition	Not at all – Extremely
9	Right now, how calm do you feel?	Stress	Not at all – Extremely
10	Right now, how much energy do you have?	Fatigue	None at all – Full of energy
11	My sleep last night was:	Sleep	Light sleep – Deep sleep
12	Last night, the first time I got to sleep, I:	Sleep	Just never could fall asleep – Fell asleep almost immediately
13	Last night I was:	Sleep	Awake all night long – Awake very little
14	Last night, when I woke up or was awakened, I:	Sleep	Couldn't get back to sleep – Got back to sleep immediately
15	I would describe my sleep last night as:	Sleep	A bad night's sleep – A good night's sleep

Abbreviations: VAS: visual analog scale.

Table S4: Afternoon survey (14:00–15:00).

No.	Question	Construct	Anchors (VAS 1–100)
1	Right now, how sad do you feel?	Depressed mood	Not at all – Extremely
2	Right now, how anxious do you feel?	Anxiety	Not at all – Extremely
3	Right now, how sharp is your thinking?	Cognition	Not at all – Extremely
4	Right now, how stressed do you feel?	Stress	Not at all – Extremely
5	Right now, how fatigued do you feel?	Fatigue	Not at all – Extremely
6	Right now, how hopeful do you feel about things?	Depressed mood	Not at all – Extremely
7	Right now, how tense do you feel?	Anxiety	Not at all – Extremely
8	Right now, how difficult is concentrating?	Cognition	Not at all – Extremely
9	Right now, how calm do you feel?	Stress	Not at all – Extremely
10	Right now, how much energy do you have?	Fatigue	None at all – Full of energy

Abbreviations: VAS: visual analog scale.

medRxiv preprint doi: https://doi.org/10.64898/2026.01.26.26344234; this version posted March 9, 2026. The copyright holder for this preprint (which was not certified by peer review) is the author/funder, who has granted medRxiv a license to display the preprint in perpetuity.
It is made available under a CC-BY 4.0 International license .

Table S5: Evening survey (20:00–21:00).

No.	Question	Construct	Anchors (VAS 1–100)
1	Right now, how sad do you feel?	Depressed mood	Not at all – Extremely
2	Right now, how anxious do you feel?	Anxiety	Not at all – Extremely
3	Right now, how sharp is your thinking?	Cognition	Not at all – Extremely
4	Right now, how stressed do you feel?	Stress	Not at all – Extremely
5	Right now, how fatigued do you feel?	Fatigue	Not at all – Extremely
6	Right now, how hopeful do you feel about things?	Depressed mood	Not at all – Extremely
7	Right now, how tense do you feel?	Anxiety	Not at all – Extremely
8	Right now, how difficult is concentrating?	Cognition	Not at all – Extremely
9	Right now, how calm do you feel?	Stress	Not at all – Extremely
10	Right now, how much energy do you have?	Fatigue	None at all – Full of energy

Abbreviations: VAS: visual analog scale.

Table S6: Exit survey.

No.	Question	Construct	Anchors (Likert scale 1–5) / Type
1	I think that I would like to use this system frequently.	SUS	Strongly disagree – Strongly agree
2	I found the system unnecessarily complex.	SUS	Strongly disagree – Strongly agree
3	I thought the system was easy to use.	SUS	Strongly disagree – Strongly agree
4	I think that I would need the support of a technical person to be able to use this system.	SUS	Strongly disagree – Strongly agree
5	I found the various functions in this system were well integrated.	SUS	Strongly disagree – Strongly agree
6	I thought there was too much inconsistency in this system.	SUS	Strongly disagree – Strongly agree
7	I would imagine that most people would learn to use this system very quickly.	SUS	Strongly disagree – Strongly agree
8	I found the system very awkward to use.	SUS	Strongly disagree – Strongly agree
9	I felt very confident using the system.	SUS	Strongly disagree – Strongly agree
10	I needed to learn a lot of things before I could get going with this system.	SUS	Strongly disagree – Strongly agree
11	Did using this system help you become more aware of connections between your behaviors, feelings, and your health? If so, how?	Patient feedback	Free response
12	If you had access to a tool like this in your everyday life (outside the hospital), how do you think it would help you manage your epilepsy?	Patient feedback	Free response
13	Are there any other features you would like to see added?	Patient feedback	Free response
14	Feel free to share any other feedback, thoughts, or suggestions.	Patient feedback	Free response

Abbreviations: SUS: System Usability Scale.
